# Mucosal Inflammation as a Driver of Gd-IgA1 Production in IgA Nephropathy: Mechanistic Support for Targeted Therapies

**DOI:** 10.64898/2026.10.19.759132

**Published:** 2026-10-24

**Authors:** Katerina Zachova, Alica Cutkova, Petr Kosztyu, Yukako Ohyama, Kazuo Takahashi, Josef Zadrazil, Jiri Orsag, Nadezda Petejova, Dana Galuszkova, Jiri Mestecky, Milan Raska

## Abstract

Rationale & Objective: Immunoglobulin A nephropathy (IgAN) is characterized by mesangial deposition of immune complexes containing galactose-deficient IgA1 (Gd-IgA1), predominantly with λ light chains and Gd-IgA1 glycans-recognizing antibodies. Disease exacerbations are frequently linked to mucosal infections, underscoring the role of dysregulated mucosal immunity. Whereas current therapeutic strategies targeting Gd-IgA1-producing cells are largely evaluated based on clinical endpoints, a critical gap remains in understanding how these therapies influence Gd-IgA production and whether targeting mucosal immunity represents an effective therapeutic strategy. Here, we provide a mechanistic insight into Gd-IgA1+ B cell behavior within inflammatory mucosal-like microenvironment. Study Design: Case-control study with ex vivo functional analysis. Setting & Participants: Peripheral blood mononuclear cells from patients with biopsy-proven IgAN and healthy controls. Exposure(s): Stimulation with a cytokine combination mimicking mucosal immune activation (IL-6, IL-21, BAFF, APRIL). Outcome(s): Differentiation of B cell subsets involved in IgAN and production of IgA, Gd-IgA1, and IgG. Analytical Approach: Flow cytometry, ELISA, cytometric bead array, and Western blotting. Results: Mucosal cytokine stimulation induced robust differentiation of B cells into CD38high plasmablasts and CD138high plasma cells, with a disproportionate expansion of Gd-IgA1⁺ populations in IgAN. Compared with controls, PBMCs from IgAN patients demonstrated markedly increased production of Gd-IgA1, predominantly in polymeric form and strongly enriched for λ light chains (~90% vs ~51%). This response was accompanied by selective expansion of Gd-IgA1⁺λ⁺ plasma cells, identifying a disease-specific, highly responsive B cell compartment. Limitations: The cytokine combination was selected to reflect key signals present in mucosal immune environments, although in vivo conditions are more complex. Conclusions: Mucosal cytokine signals directly amplify pathogenic B cell responses in IgAN, driving production of polymeric, λ-enriched Gd-IgA1. These findings provide mechanistic validation for therapies targeting the mucosal-systemic axis and offer a biologic explanation for the clinical efficacy of gut-directed corticosteroids and BAFF/APRIL inhibition.

## Full Text Availability

The license terms selected by the author(s) for this preprint version do not permit archiving in PMC. The full text is available from the preprint server.

